# Automated Entire Thrombus Density Measurements for Robust and Comprehensive Thrombus Characterization in Patients with Acute Ischemic Stroke

**DOI:** 10.1371/journal.pone.0145641

**Published:** 2016-01-14

**Authors:** Emilie M. M. Santos, Wiro J. Niessen, Albert J. Yoo, Olvert A. Berkhemer, Ludo F. Beenen, Charles B. Majoie, Henk. A. Marquering, MR CLEAN investigators

**Affiliations:** 1 Dept. of Radiology, Erasmus MC, Rotterdam, the Netherlands; 2 Dept. of Medical Informatics, Erasmus MC, Rotterdam, the Netherlands; 3 Dept. of Radiology, AMC, Amsterdam, the Netherlands; 4 Dept. of Biomedical Engineering and Physics, AMC, Amsterdam, the Netherlands; 5 Faculty of Applied Sciences, Delft University of Technology, Delft, the Netherlands; 6 Department of Radiology, Division of Interventional Neuroradiology, Texas Stroke Institute, Plano, Texas, United States of America; INSERM U894, FRANCE

## Abstract

**Background and Purpose:**

In acute ischemic stroke (AIS) management, CT-based thrombus density has been associated with treatment success. However, currently used thrombus measurements are prone to inter-observer variability and oversimplify the heterogeneous thrombus composition. Our aim was first to introduce an automated method to assess the entire thrombus density and then to compare the measured entire thrombus density with respect to current standard manual measurements.

**Materials and Method:**

In 135 AIS patients, the density distribution of the entire thrombus was determined. Density distributions were described using medians, interquartile ranges (IQR), kurtosis, and skewedness. Differences between the median of entire thrombus measurements and commonly applied manual measurements using 3 regions of interest were determined using linear regression.

**Results:**

Density distributions varied considerably with medians ranging from 20.0 to 62.8 HU and IQRs ranging from 9.3 to 55.8 HU. The average median of the thrombus density distributions (43.5 ± 10.2 HU) was lower than the manual assessment (49.6 ± 8.0 HU) (p<0.05). The difference between manual measurements and median density of entire thrombus decreased with increasing density (r = 0.64; p<0.05), revealing relatively higher manual measurements for low density thrombi such that manual density measurement tend overestimates the real thrombus density.

**Conclusions:**

Automatic measurements of the full thrombus expose a wide variety of thrombi density distribution, which is not grasped with currently used manual measurement. Furthermore, discrimination of low and high density thrombi is improved with the automated method.

## Introduction

Fast restoration of blood flow is key to successful acute ischemic stroke treatment. The American Heart/Stroke association recommends intra-venous thrombolysis with alteplase, as an early treatment after the exclusion of intracranial hemorrhage on non-contrast CT (NCCT) [1]. However some thrombi fail to dissolve and for these patients intra-arterial treatment should be considered [2]. A main challenge in current ischemic stroke management is the selection of patients in which either intra-arterial or intravenous treatment would be advantageous. Recent research shows that thrombus length measured on NCCT (>8mm) is a potential predictor of Intra-venous Thrombolysis (IVT) failure [3]. However, commonly smaller thrombi do not dissolve after administration of IVT and therefore thrombus length has limited value in predicting thrombolysis success. Understanding the composition of the thrombus may provide additional information to support determination of the most promising treatment. For this goal, thrombus composition has been studied by means of its density values on CT imaging[4].

Studies have related the presence of an hyperdense artery sign—a visual mark of intraluminal thrombus on non-contrast CT- with success of restoring cerebral perfusion, stroke severity, and functional outcome [5–15]. Although the specificity of detecting hyperdense thrombi is high, its sensitivity is rather low [16]. As an alternative to the qualitative classification of hyperdense artery sign, it has been proposed to quantitatively measure thrombus density. This measurement can be performed by placing one or multiple small regions of interest (ROIs) in the thrombus on NCCT–for example using measuring tools available on diagnostic workstations [17–19]. Some studies have successfully related thrombus density with success of restoring cerebral perfusion, stroke severity, and functional outcome. However, other studies did not find such an association [20,21].

The drawback of the current manual thrombus density assessment using small ROIs is that this procedure is prone to inter-observer variability and observer bias [22]. Furthermore, due to the small size of the ROIs with respect to the thrombus volume, this measurement is sensitive to partial volume effects, noise and may be highly variable due to the heterogeneous thrombus composition [23]. To circumvent this, we developed an automated segmentation method to segment the thrombus in CTA images [24]. The method performs automated density measurements of the entire thrombus rather than characterizing this thrombus with a single value. In this study, we investigate the additional information provided by entire thrombus density assessment and evaluate its agreement with current standard manual measurements.

## Material and Methods

### Patient Selection

Baseline NCCT and CT Angiography (CTA) image data sets of Three-hundred-eighty-eight patients with a clinical diagnosis of acute ischemic stroke due to proximal intracranial arterial occlusion were retrospectively collected in the multi-center image database of the MR CLEAN clinical trial [2]. Following guidelines [25] indicating that for accurate thrombus representation slice thickness smaller or equal to 2.5 mm are required, datasets with slice thickness larger than 2.5 mm (n = 199) were excluded. Further exclusion criteria were low image quality due to movement artefacts (n = 39) or extensive noise on either NCCT or CTA (n = 3), poor contrast (n = 3), a scan range not including the complete intracranial artery tree (n = 4), and scans without visible contralateral vessel (n = 2) assessed by at least one expert observer. It is common practice to initiate IV thrombolysis after completion of the NCCT, which may alter the thrombus [26]. To avoid bias due to thrombus change between NCCT and CTA, patients with a time difference between CTA and NCCT imaging exceeding 30 minutes and patients that showed a difference in the thrombus position or size on the two imaging modalities were also excluded (n = 3). The remaining 135 patients were included in this study. Detailed information about the scanners, scanning protocol, and reconstruction parameters can be found in **Table 1**.

**Table 1:** Information about the scanners, scanning parameters, and reconstructions parameters of the scanners used in this study.

	n patients
**Scanner manufacturer and model**	
**GE MEDICAL SYSTEMS**	**13**
LightSpeed VCT	13
**PHILIPS**	**24**
Brilliance 40	10
Brilliance 64	5
iCT 256	9
**SIEMENS**	**52**
Biograph 64	2
Sensation 64	22
SOMATOM Definition AS+	3
SOMATOM Definition Flash	25
**TOSHIBA**	**46**
Aquilion ONE	46
**Tube voltage (KVP)**	
100	2
120	133
**Slice Thickness reconstruction (mm)**	
≤0.5	75
≤0.75<	24
≤1<	27
≤1.5<	6
≤2	3
**Convolution kernel types**	
FCXX*	20
FLXX*	26
HXXx*	45
J45s\X*	7
SOFT	13
UA	2
UB	22
**Filter types**	
none	29
FLAT	28
LARGE	26
MEDIUM	19
SMALL	9
UA	2
UB	22

*Kernel type details: FC22, FC25, FC26, FC27, FC28, FC29, FC30, FC31, FC32, FC33, FC34, FC35, FC36, FC69, FC70, FC71, FC72, FL01, FL02, FL03, FL04, FL05, FL06, FL07, FL08, FL09, FL10, FL11, FL12, FL13, FL14, FL15, FL16, FL17, FL18, FL19, FL20, FL21, FL22, FL23, H30f, H31s, H41s, H60s, H70h, J45s\10, J45s\11, J45s\4, J45s\5, J45s\6, J45s\7, J45s\8, J45s\9.

### Ethic statement

The MR CLEAN study protocol was approved by the Medical and Ethical Review Committee (Medisch Ethische Toetsings Commissie of the Erasmus MC, Rotterdam, The Netherlands) and the research board of each participating center (Academic Medical Center Amsterdam; Maastricht University Medical Center, Maastricht; Sint Antonius Hospital, Nieuwegein; Leiden University Medical Center, Leiden; Rijnstate Hospital, Arnhem; MC Haaglanden, the Hague; HAGA Hospital, the Hague; University Medical Center Utrecht; Radboud University Medical Center, Nijmegen; Sint Elisabeth Hospital, Tilburg; Isala Klinieken, Zwolle; Reinier de Graaf Gasthuis, Delft; VU Medical Center, Amsterdam; University Medical Center Groningen; Atrium Medical Center, Heerlen; Catharina Hospital, Eindhoven; Medical Spectrum Twente, Enschede; Sint Lucas Andreas Hospital, Amsterdam; all in the Netherlands). All patient records and images were anonymized prior to analysis. All patients or legal representatives signed informed consent.

### Automated Density Measurement of entire thrombi

For density measurement of the entire thrombus an automated thrombus segmentation method as previously described [24] was used. This method segments the thrombus in CTA images in three steps: (1) segmentation of the contralateral vasculature using 2 manually placed seeds points by a trained observer (ES); (2) segmentation of the occluded artery by mapping the contralateral segmentation to the occluded artery using mirror symmetry; (3) thrombus segmentation using intensity based region growing. By performing a rigid registration of the CTA and NCCT scans (Elastix®[27], University Medical Center Utrecht, The Netherlands), the CTA-based thrombus segmentation was automatically projected on NCCT. If visual inspection revealed that the thrombus segmentation was suboptimally registered, a correction could be performed using manual rigid registration tools (developed in Mevislab®[28], MeVis Medical Solutions AG, Fraunhofer MEVIS, Bremen, Germany). Finally, the density distribution of the entire thrombus was sampled in the entire thrombus on NCCT, and thrombus volume was measured in mm^3^.

### Manual Thrombus Density Measurement

**Fig 1:**
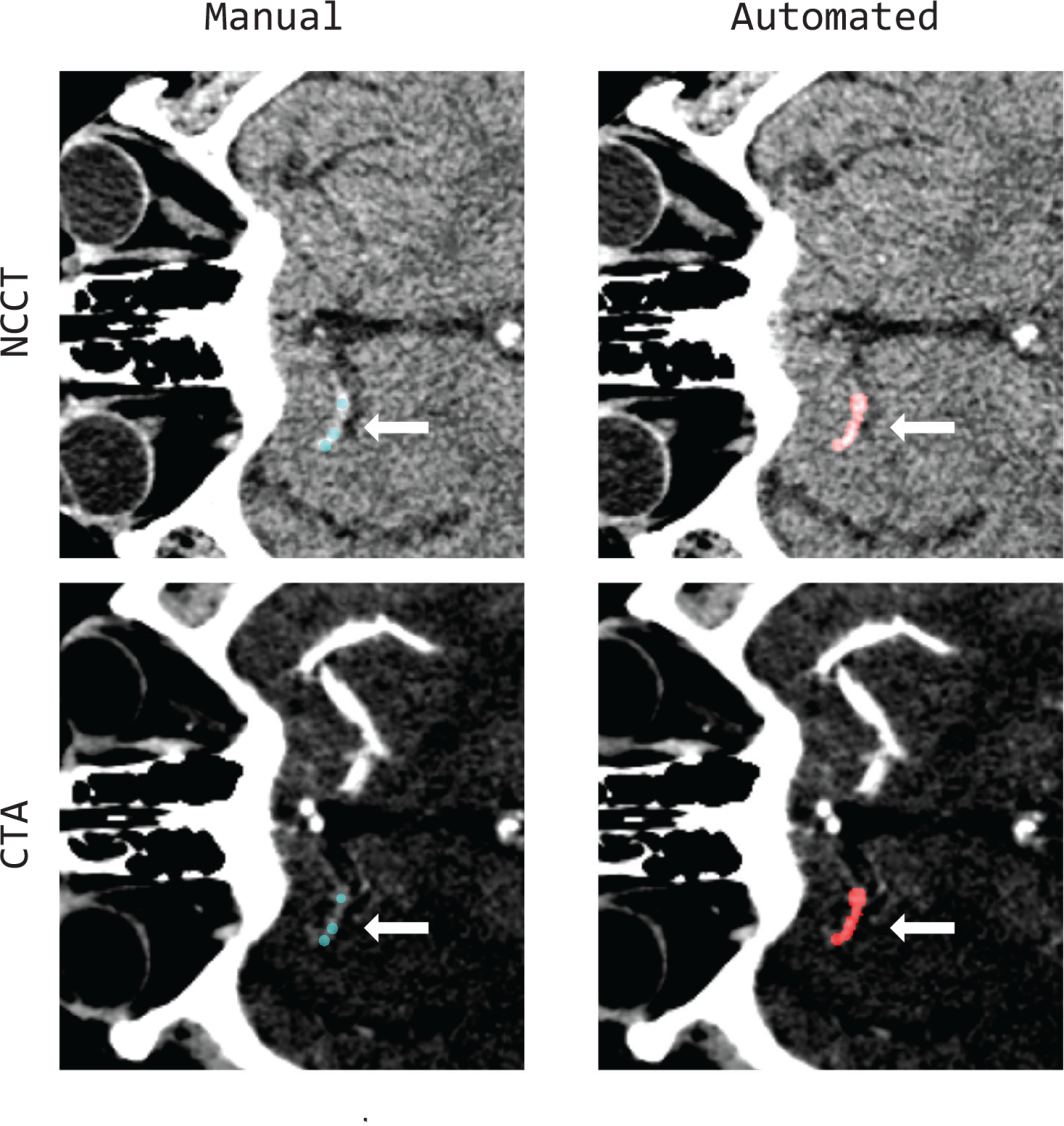
Example of registered NCCT (left column) and CTA (right column) images of a patient suffering from an M1 occlusion (white arrows). The green overlay (on the left) represents the manually measured density using the three spherical ROI. On the right panel, the red overlay highlights the result of the automated thrombus segmentation on CTA.

Manual thrombus density measurements were performed as previously described[22]. In short, three standardized small spherical regions of interest were placed simultaneously within the thrombus on the registered CTA and NCCT images using in-house developed annotation software (**Fig 1**). Subsequently, absolute thrombus densities were determined. The previously described annotations were performed by three neuroradiologists (AY, LB and CM), each with more than 10 years of experience. Observer 1, as reference observer, measured all 135 patients, Observer 2 and 3 measured respectively 77 and 61 patients for interobserver agreement determination. They only had access to baseline NCCT and CTA images during thrombus assessment and were blinded from measured density and all clinical information.

### Statistical Analysis

The density distribution of the entire thrombus was represented with median, interquartile range (IQR), skewedness, and kurtosis. Normality of the distribution was assessed using the Shapiro-Wilk test. The average density values, standard deviation (SD), and the range of the medians of all the density distributions were determined. Associations between the median density, volume, and IQR of the entire thrombus were determined by calculation of the Pearson’s correlation coefficient (PCC). The manual measurements resulted in one average density value per thrombus per observer. Mean, SD, and range was used to describe all the variation of these manual measurements. Interobserver agreement was assessed using Bland-Altman analysis and the Intraclass correlation coefficient (ICC). To assess the agreement between manual measurements and the density distribution of the entire thrombus, we compared the manual measurements with the median and IQR of the measurement of the entire thrombus using the same statistical analysis. Difference in mean and median intensities was investigated using a paired t-test. Subsequently, the association of manual measurements with thrombus volume and density spread (IQR) was investigated using scatter plots and calculation of the PCC. To assess potential bias due to variations in slice thickness and reconstruction convolution kernel on measured density, (1) correlations between slice thickness and thrombus median density was assessed by the calculation of the PCC and (2) differences between the average median density per reconstruction kernel was evaluated using a non-parametric ANOVA. Significance level was set to p-value ≤ 0.05. All analyses were performed using IBM SPSS Statistics software, version 20.0 (IBM Corporation, Armonk, NY, USA).

## Results

The average age was 65 (± 13) years and 63% (86) of the patients were male. The occlusion site was in the Internal Carotid Artery (ICA) and ICA-Terminus in 24% of the cases (n = 32), in the middle cerebral arteries segment M1 in 64% (n = 87) of the cases, and in the M2 in 12% (n = 16) of the cases. Automated thrombus segmentation was successful for all patients. Twenty out of 135 (15%) patients required additional manual correction of the CTA to NCCT registration.

**Fig 2:**
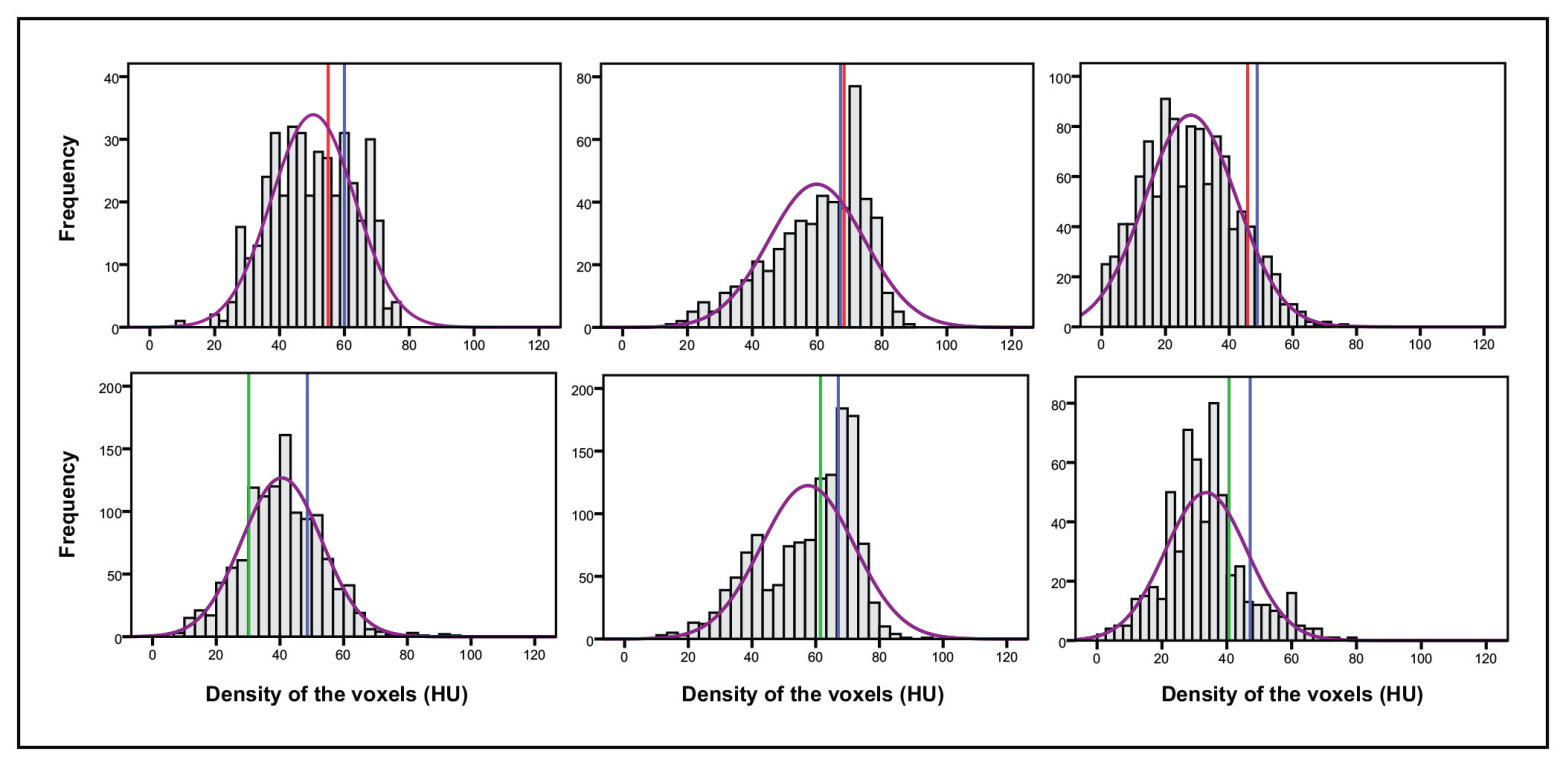
Histograms of density distribution of six automatically segmented entire thrombi. The colored vertical lines indicate the observer density measurements (blue for observer 1, red for observer 2 and green for observer 3). The purple curve represents the fitted normal curve.

Examples of density distributions resulting from entire thrombus segmentations for six patients are provided in **Fig 2**. All thrombus characteristics are presented in **Table 2** and in the supporting information dataset 1 (**S1 Dataset**). For the density distribution of the entire thrombus, the average mean and median were 42.9 ± 9.0 HU and 43.4 ± 10.2 HU respectively. The average thrombus volume was 52 ± 38 mm^3^, and the average IQR width was 20.3 ± 7.4 HU. In nineteen percent (n = 26) of the thrombi, the density was normally distributed.

**Table 2:** Descriptive statistics of the assessment of entire thrombus and the manual density measurements.

	Average	SD	Min	Max
Density (in HU) and Volume (in mm3) of the entire thrombi (n = 135)				
Mean	42.9	9.0	20.3	61.7
Median	43.4	9.9	20.0	64.0
SD	13.6	3.4	6.5	24.3
Minimum	9.3	9.9	0.0	37.0
Maximum	75.1	12.0	45.0	120.0
Interquartile Range	30.3	7.4	9.3	55.8
Skewedness	-0.1	0.4	-1.3	2.1
Kurtosis	-0.3	0.7	-1.4	4.6
Volume	52.2	38.1	5.0	189.6
Density (in HU) of the manual assessments				
Observer 1 (n = 135)	51	7.5	26.5	71.8
Observer 2 (n = 77)	50.4	8.1	24.3	72.2
Observer 3 (n = 61)	45.5	7.6	28.0	61.5
All Observers (n = 273)	49.6	8.0	24.3	72.2

**Fig 3:**
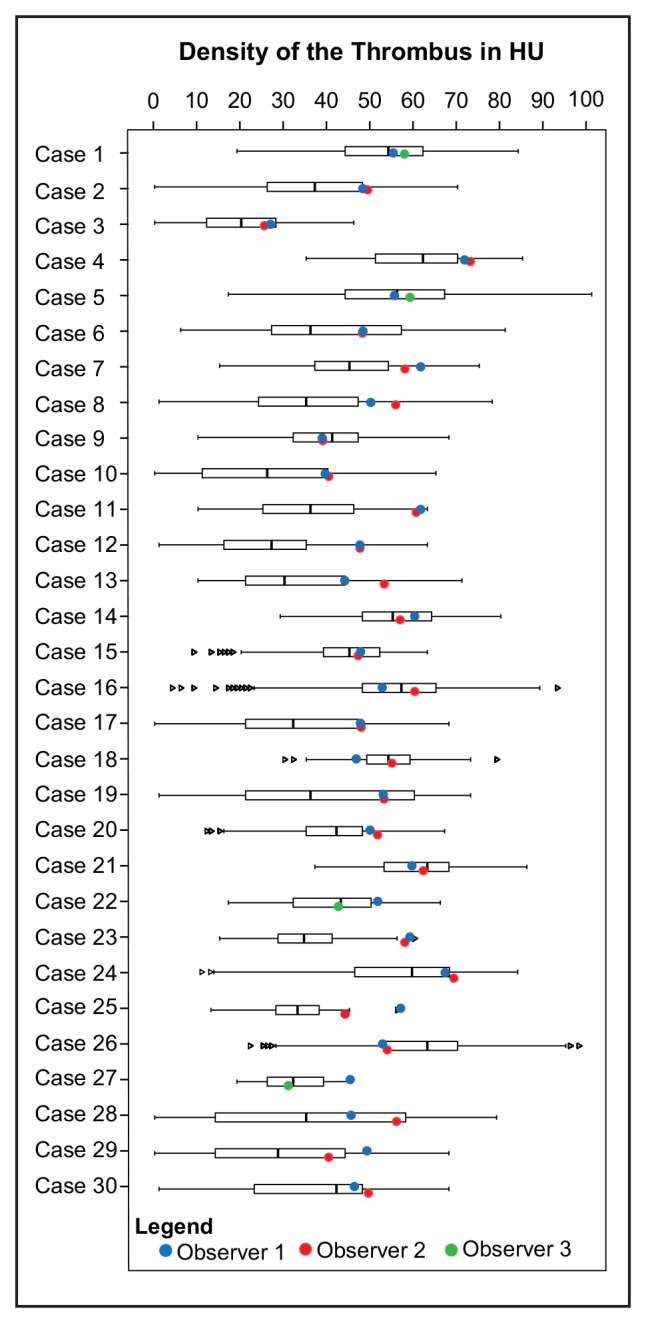
Density distributions of a randomly selected set of 30 patients and corresponding manual measurements. The boxplots represent the first to third quartile the density distribution of the entire thrombus segmentation. The vertical lines indicate the variability outside the Q1 and Q3. The colored circles indicate the observer density measurements (blue for observer 1, red for observer 2 and green for observer 3).

**Fig 4:**
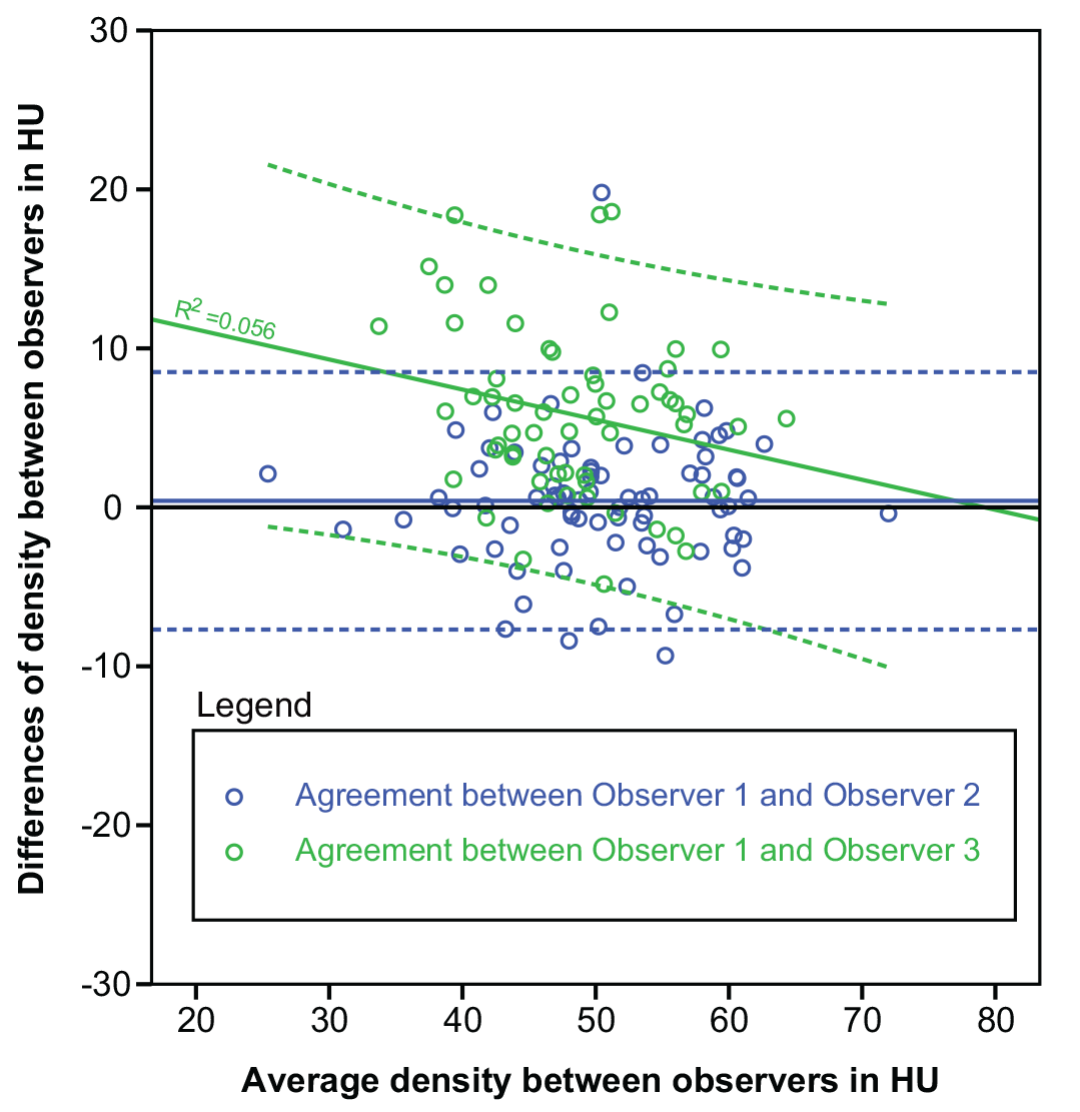
Bland-Altman graph assessing the Interobserver agreements of Observer 2 and Observer 3 with the reference Observer 1. The colors indicate pairs of observers (blue for observer 1 and observer 2, and green for observer 1 and observer 3). The color dotted lines represent the limits of agreements; the solid line represents the mean paired difference.

**Fig 5:**
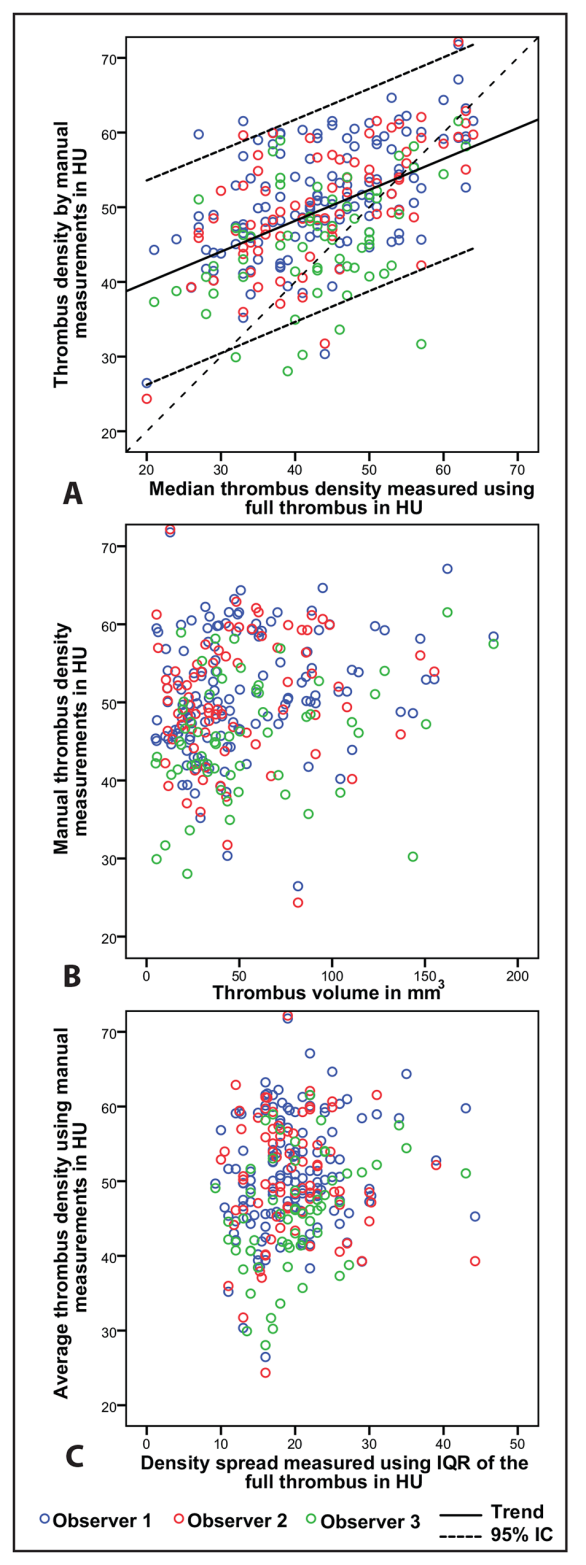
Scatter plot of the manual thrombus density measurement compared to the thrombus measured of the entire thrombus (A), Scatter plot of the manual thrombus density measurement compared to the thrombus volume(B). Scatter plot of the manual thrombus density measurement compared to the thrombus density IQR (C). The colors indicate the different observers (blue for observer 1, red for observer 2, and green for observer 3).

No statistically significant association was found between the mean density values and the thrombus volume and the spread (SD) of the density measures (**Table 3**). Manually assessed thrombus density, with an average value of 49.6 ± 8.0 HU was significantly higher than the median (Δ = 6.2 HU) and mean (Δ = 6.7 HU) value of the automatically derived density distributions (p < 0.05). **Fig 3** shows box-plots of 30 randomly selected patients with their corresponding manual measurements. The original voxel density measures for this set are available in the supporting information dataset 2 (**S2 Dataset**). Interobserver analysis (**Fig 4**) revealed an ICC of 0.87 between observer 1 and 2, and an ICC of 0.72 for observer 1 and 3. Regression analysis showed an agreement between the manual measurement of the reference observer and the average density of the thrombus of a constant value of 11.7 (95%CI: 5.3–18.1) HU and a slope of 0.64 (95%CI: 0.50–0.80), a PCC of 0.52, and a R^2^ of 0.29. Statistically significant associations were found between the reference manual density measurements and the thrombus IQR and thrombus volume; however the coefficient of determination were very small with R^2^ of 0.03 and 0.04 respectively (**Fig 5** and **Table 3**). There was no statistically significant correlation between slice thickness and measured median density of the thrombus (PCC = 0.067, p = 0.44). The non-parametric ANOVA analysis showed no significant differences between the different convolution kernels (p = 0.35).

**Table 3:** Results of statistical analyses relating manual thrombus density measurements and median value of the entire thrombus measurements with the heterogeneity of the thrombus, as expressed by the inter-quartile range (IQR) of the entire thrombus measurement and with the thrombus volume in mm^3^.

Statistical analysis	Thrombus IQR (HU)	Thrombus volume (mm3)
Manual thrombus measurement (Average)		
**Slope**	0.14*	0.04*
**Intercept**	12.6	47.6
**PCC**	0.17	0.19
**R**^**2**^	0.03	0.04
Entire thrombus measurement (Median)		
**Slope**	-0.003	-0.002
**Intercept**	20.6	43.6
**PCC**	0.07	0.06
**R**^**2**^	0.05	0.001

* p ≤ 0.05

## Discussion

We have introduced an automated method for entire thrombus density assessment on CT, which provides information of the whole thrombus density distribution rather than representing the thrombus density with a single value, as obtained with the current manual measurement. Our study showed large variation in the density histograms among thrombi, indicating that entire thrombus measurement provides more information than the conventional measurements. Notably, the comparison of the entire thrombus density distributions with the manual measurements has shown that manual measurements of low-density thrombi are overestimated compared to high-density thrombi. This decreases differences in intensity measurements between low and high dense thrombus, thereby reducing its discriminating power. Consequently, the use of entire thrombus density measurements may improve the distinction of low from high dense thrombi.

Usually, thrombus density is assessed by visual inspection (e.g., hyperdense artery sign) [16]. Quantitative assessment of thrombus density was introduced as support to visual inspection [29]. These quantitative measurements established the relation between thrombus density and thrombus composition derived from histology: platelet-rich thrombi were found to have lower densities on NCCT than red blood cell-rich thrombi [4,30]. The entire thrombus density distribution assessment as presented in this study, determines the range of densities within a single thrombus and could conceivably be used to measure the heterogeneity of the thrombus composition. Because the ratio of thrombus components (e.g. red and white blood cells, fibrin, debris) influences the thrombus response to fibrinolysis [31], retrieving information on the composition from baseline imaging could allow a better identification of patients who are likely to benefit from IVT.

We found that manual thrombus density assessments were significantly higher than the median density measured of the whole thrombus. This bias was negatively correlated with median thrombus density. This implies that for a thrombus with a low density, the manually measured density is overestimated. This overestimation of low dense thrombi reduces the density difference between low- and high-density thrombi. As a consequence, this reduced difference in measured density may explain why strong association of thrombus density with treatment success that was found some studies [19] could not be confirmed in other studies [20,21].

Our study has some limitations. First, there is a lack of a gold standard for evaluation of the accuracy of the thrombus density measurements. As such, the accuracy of the automated method could not be determined. Instead, we compared the difference between the median of the density distribution measured by the automated method with manual measurements. However, this measurement is prone to interobserver variation most likely due to variation in composition within the thrombus [23]. On the other hand, a more extensive manual measurement of the thrombus could be performed; for example, perform a complete manual segmentation of the thrombus (e.g. using a combinations of the segmentation of the thrombus in every slices [19] or using a multiple step manual delineation method[24]) such that measurement bias due to the thrombus heterogeneity would be reduced. However, to our knowledge, these more extensive methods are hardly applicable to low–dense thrombi. Alternatively, validation of the density measures could be performed by comparison with histological analysis of retrieved thrombus[4,18]. However, these histological analyses of retrieved thrombus were not available and therefore such a comparison could not be performed. Interobserver agreement was good. Due to the indistinct borders of some thrombi, it is possible that a part of stagnant blood was included in the segmentation and consequently in the thrombus density measurements, leading to a biased estimation of the thrombus density. The automated segmentation method used in our study was design to avoid inclusions of stagnant blood with the use of a finely tuned intensity based region growing segmentation [24], which was validated using manually segmented thrombi on NCCT. The validation suggested that, at most, only a minimal amount of unclotted blood was included into the thrombus segmentation by the automated method.

Forty-one out of 197 scans had movement artefacts on CTA or NCCT, preventing accurate registration, which is a prerequisite for the automated method. This limits the applicability of the automated approach in clinical practice. Two patients had multiple contralateral occlusions, hampering the automated segmentation. As a consequence, in our population of patients with proximal large vessel occlusions of the anterior circulation, the automatic density measurement could not be used for seventeen percent (17%) of the patients. Due to the imbalanced availability of our expert observers (Observer 2 and 3) during the assessment of the thrombus density, the measurements were unequally divided amongst the observers. However, we do not expect that this has influenced interobserver analysis and comparison with the full thrombus measurements[32]. It is expected that an entire thrombus measurement is more sensitive to partial volume effects since the edges of the thrombus are also included in the measurements. Partial volume effects result in an underestimation of high density signal, particularly for small thrombi. However we could not find a significant association between measured median density and thrombus volume. Moreover, no significant correlation between measured median and slice thickness was found, suggesting that partial volume effect due to slice thickness reconstruction was not very severe in our analysis. The applied reconstruction kernels may influence measured thrombus densities; however, in this study no significant differences were found, implying that no correction was necessary. As this study is a retrospective analysis of a prospective trial[2], further studies should be performed to demonstrate the feasibility of this method in acute clinical setting. Finally, although entire thrombus density measurement gives a much more extensive estimation of thrombus density information, the therapeutic implications was beyond the scope of this study. Further analysis will be performed to evaluate entire thrombus density associations with treatment efficiency. Accurate information on thrombus density is important for assessing its association with outcome or treatment success [31]. With an increasing number of available treatment options, precise characterization of the target is required to enable optimal treatment selection for a given type and size of thrombus[33]. Although density measurement of entire thrombus requires supplementary resources (software and/or processing time), we believe that the resulting thrombus characterization would support better AIS management.

## Conclusion

Our study exposed large variations in shapes of density histograms in thrombi, indicating that currently used manual measurement result in oversimplification of thrombus characteristics. Automated density measurements of entire thrombi have the potential to enhance the discrimination between low and high dense thrombi and consequently may guide treatment decision and predict responsiveness. Further multicentre studies on larger cohorts are necessary to confirm our results in other populations.

## Supporting Information

S1 Dataset Dataset containing all measured thrombi characteristics and corresponding manual density measures. The contained characteristics are volume, mean, median, standard deviation, skew, kurtosis and interquartile range.
(SAV)

S2 Dataset Dataset containing the all the densities in HU of each voxel constituting the thrombi of the example set of 30 patients. (SAV)
